# Lung Cancer Prediction Using Neural Network Ensemble with Histogram of Oriented Gradient Genomic Features

**DOI:** 10.1155/2015/786013

**Published:** 2015-02-23

**Authors:** Emmanuel Adetiba, Oludayo O. Olugbara

**Affiliations:** ICT and Society Research Group, Durban University of Technology, P.O. Box 1334, Durban 4000, South Africa

## Abstract

This paper reports an experimental comparison of artificial neural network (ANN) and support vector machine (SVM) ensembles and their “nonensemble” variants for lung cancer prediction. These machine learning classifiers were trained to predict lung cancer using samples of patient nucleotides with mutations in the epidermal growth factor receptor, Kirsten rat sarcoma viral oncogene, and tumor suppressor p53 genomes collected as biomarkers from the IGDB.NSCLC corpus. The Voss DNA encoding was used to map the nucleotide sequences of mutated and normal genomes to obtain the equivalent numerical genomic sequences for training the selected classifiers. The histogram of oriented gradient (HOG) and local binary pattern (LBP) state-of-the-art feature extraction schemes were applied to extract representative genomic features from the encoded sequences of nucleotides. The ANN ensemble and HOG best fit the training dataset of this study with an accuracy of 95.90% and mean square error of 0.0159. The result of the ANN ensemble and HOG genomic features is promising for automated screening and early detection of lung cancer. This will hopefully assist pathologists in administering targeted molecular therapy and offering counsel to early stage lung cancer patients and persons in at risk populations.

## 1. Introduction

The entire human cells, which depend heavily on regular and adequate supply of oxygen to function effectively, may suffer in the event of impairment in oxygen inflow. The lung is the place where the required oxygen is taken in and excess carbon-dioxide, which can be toxic to the body, is released. Lungs purify air intake using cleaning systems, which destroy harmful substances that travel in the air [1–3]. Cilia, the tiny hairs that line the bronchi in the lungs have mucus, which moves foreign objects such as bacteria and viruses out and this provides the first defensive mechanism in the lungs. However, because the lungs are delicate organs and constantly exposed to the external environment, they are prone to a range of illnesses generally referred to as lung diseases. Some of these diseases are lung cancer, chronic obstructive pulmonary disease, emphysema, asthma, chronic bronchitis, pneumonia, pulmonary fibrosis, sarcoidosis, and tuberculosis. Lung cancer develops as a result of a sustained genetic damage to normal lung cells, which consequently lead to an uncontrolled cell proliferation. It is also called bronchiogenic carcinoma and it mostly starts in the cells lining the bronchi of the lungs [1]. Smoking is responsible for about 85% of lung cancer, but there are empirical evidences that arsenic in water and beta-carotene supplement also increase the predisposition to the disease. Other lung cancer carcinogens include asbestos, radon gas, arsenic, chromium, nickel, polycyclic aromatic hydrocarbons, or genetic factor [4].

In classical oncology, lung cancer is named based on how the cancerous cells look under a microscope. The two major histological subtypes of lung cancers are small cell lung cancer (SCLC), which is approximately 13% and non-small cell lung cancer (NSCLC) that constitutes about 87% of the disease. NSCLC subtype is more dangerous because it spreads more slowly than SCLC [5, 6]. Using the tissue, node, and metastasis (TNM) classification system, NSCLC is divided into four stages, which include Stage I, Stage II, Stage III, and Stage IV. Stage III is the most sophisticated of the different stages because it includes a tumor that has metastasized into the chest wall, diaphragm, and pleura of the mediastinum or heart. It has mediastinum lymph node involvement and it is often impossible to remove the cancerous tissues with the degree of spread at this stage. The prognosis statistics of NSCLC show that five-year overall survival of patients with stage IA is 67% and for patients with stage IIA, it is 55%. Patients with stage IIIA have 23% survival chance of five years after surgery while patients with stage IV only have 1% [5, 7, 8].

Lung cancer, like other cancers, is a highly complex and heterogeneous genetic disease. Researchers have identified two major categories of genes that suffer mutations and genetic alterations of diverse kinds in lung cancer cells. These categories are oncogenes and tumor suppressor genes. Some examples of the oncogenes are epidermal growth factor receptor (EGFR), Kirsten rat sarcoma viral oncogene (KRAS), MYC, and BCL-2, and common examples of tumor suppressor genes are tumor suppressor p53 (TP53) and retinoblastoma (RB) [9–13]. As recent as 2013, Chen et al. [14] carried out a study to identify genes that carry somatic mutations of various types in lung cancer and reported 145 genes with high mutation frequencies. The study established that the three most frequently mutated genes in lung cancer are EGFR, KRAS, and TP53 with mutation frequencies of 10957, 3106, and 2034, respectively. The authors further posited that “these frequently mutated genes can be used to design kits for the early detection of carcinogenesis”.

The classical equipment used for the detection and classification of lung tumors includes X-ray chest films, computer tomography scans (CT), magnetic resonance imaging (MRI), and Positron emission tomography (PET) [15]. The overall identification of lung cancer images using this radiological equipment is very low at the early stage of the disease [16]. This is because pathologists who interpret the radiological scans do not sometimes differentiate accurately between malignant, benign, and other forms of lesions in the lung. However, with the landmark breakthrough in the complete human genome sequencing study, there has been a gradual shift from radiographic oncology to genomic-based cancer detection [17]. This trend is highly expected because all forms of cancers emanate primarily from genomic abnormalities.

Molecular methods are therefore currently popular for genetic screening of patients to detect somatic mutations in lung cancer [18–21]. Direct sequencing of tumor sample is one of the molecular methods frequently used, but this method has been reported to have limitations such as low sensitivity, low speed, and intensive labor requirement. Assays that are based on quantitative real-time polymerase chain reaction (PCR), fluorescence* in situ* hybridization (FISH), immunohistochemistry, and microarray technologies have also been developed for detecting different classes of genomic defects. Although some of these techniques have good sensitivity, they are conversely limited in the degree of mutation coverage [18, 22, 23].

The EGFR mutation testing is another method that has been developed for lung cancer genetic test. This method is acclaimed to have good capability for mutation detection, but it is also prone to several limitations such as low sensitivity, longer turnaround time, high-quality tumor sample requirement, the need for expert experience, and limited coverage of only EGFR mutations [24]. In light of the shortcomings of the existing molecular testing methods, the authors in [18] opined that “great number of molecular biology methods and variety of biological material acquired from patients create a critical need for robust, well-validated diagnostic tests and equipment that are both sensitive and specific to mutations.”

This study is inspired by the critical need to develop equipment and/or models that can detect multiple mutations in the early stage NSCLC. Our overarching objectives are threefold. First, we want to leverage on the targeted sequencing (TS) capability of next generation sequencing (NGS) to predict NSCLC. Rather than the whole genome sequencing (WGS), which provides access to all genetic information in coding, regulatory and intronic regions of an organism, researchers are currently exploiting TS for genomic regions that best address their questions. This is currently a huge attraction for researchers in application niches such as cancer genomics, which is also called oncogenomics, pharmacogenomics, and forensic genomics [25].

Our second paramount objective is the adoption of the Voss mapping encoding technique and the comparison of histogram of oriented gradient (HOG) descriptor with local binary pattern (LBP) descriptor for efficient extraction of compact genomic features. The Voss mapping is reputed as a spectrally efficient numerical encoding method in genomic signal processing (GSP) research community while HOG and LBP are successful image descriptors for feature extraction in the digital image processing (DIP) research domain [26, 27]. In DIG, shape and texture are important primitive features for object description. The HOG feature descriptor is nominated for this study because it adequately captures the local appearance and shape of an object [28]. On the other hand, the LBP was considered for experimentation because of its capability to properly describe the texture of an image [27, 29]. These core characteristics of HOG and LBP are paramount for detecting and discriminating the varying shapes and textures of the Voss-mapped genomic features in this study.

Third, we want to experimentally compare multilayered perceptron artificial neural network (MLP-ANN) and support vector machine (SVM) ensembles as well as their nonensemble variants for genomic-based prediction of NSCLC using EGFR, KRAS, and TP53 Biomarkers. These machine learning classifiers have been reported to be effective in applications such as facial expression recognition, hyperspectral image processing, object detection, and Bioinformatics [30–32].

The computational approach being explored in this work will apparently afford the opportunity of reconfiguration. This will further allow us to incorporate additional somatic mutations and other genetic abnormalities into the prediction framework as new biomarkers and more mutation data become available. The MATLAB scripts resulting from this current work can potentially be interfaced with an NGS equipment such as an Illumina MiSeq sequencer to automate NSCLC prediction from a targeted sequence.

## 2. Materials and Methods

**Figure 1:**
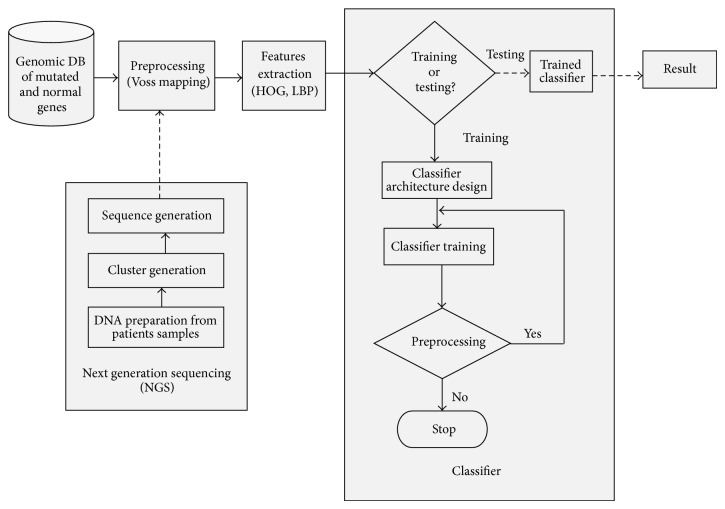
Architecture of the proposed NSCLC prediction model.

This section is a detailed report of the methods and materials utilized in this study, beginning with the architecture of the proposed NSCLC prediction model shown in Figure 1. The different phases for implementing and utilizing the model are presented as major blocks in Figure 1. The major blocks in the architecture, which include data acquisition (genomic database (DB) of mutated and normal genes), preprocessing (numerical mapping of genomic nucleotides), feature extraction, and classification are exhaustively discussed in the subsequent subsections. The next generation sequencing (NGS) block in the framework is an interface for entering test genomic sequences into the prediction model.

### 2.1. Data Acquisition

The normal nucleotides of the three genes in this study are extracts from the collaborative consensus coding sequence (CCDS) archive in the National Centre for Biotechnology Information (NCBI) repository. The CCDS project compiles identical protein annotations on the genomes of Homo sapiens (humans) and Mus musculus (mouse) using a stable identifier tagged CCDS ID. The purpose of the unique tagging is to remove the uncertainty of sequences emanating from different laboratories using different sequencing methods [33]. The CCDS IDs assigned to EGFR, KRAS, and TP53 are CCDS5514.1, CCDS8702.1, and CCDS11118.1, respectively. We used these IDs to extract the normal nucleotides of genes from the online NCBI genome repository. Mutation datasets for each of the genes were collected from the Integrated Genomic Database of Non-Small Cell Lung Cancer (IGDB.NSCLC), which is an online corpus dedicated to the archiving of NSCLC genetic defects. The somatic mutations in the corpus were, according to the authors of IGDB.NSCLC corpus, imported from COSMIC (Catalogue of Somatic Mutations in Cancer) database [34].

Deletion and substitution mutation data were extracted for both EGFR and TP53 because these two genetic events were reported in the literature to be the most predominant lung cancer somatic mutations [14, 35]. Moreover, we discovered that 99.67% of KRAS mutations in the IGDB.NSCLC database are substitution while deletion mutation data are a negligible 0.00131%. Based on these statistics, KRAS substitution mutation is also selected for this study. Overall, we acquired 6,406 samples and our experimental dataset contains six different classes, which are* normal*,* EGFR deletion*,* EGFR substitution*,* KRAS substitution*,* TP53 deletion,* and* TP53 substitution *mutations. The general statistics of the acquired data for both normal and mutated samples are shown in Tables 1 and 2.

### 2.2. Numerical Mapping of Genomic Nucleotides

The selection of a mapping method for numeric encoding of genomic sequence determines how the intrinsic properties and features of interests for a given sequence are reflected and exploited. The approaches for numerical encoding of DNA sequences are classified into fixed mapping (FM) and physicochemical property based mapping (PCPBM) [36]. The PCPBM approach involves the use of biophysical and biochemical properties of DNA molecules for sequence mapping. The methods in this category are applied in detecting biological principles and structures in DNA. Examples of PCPBM methods in the literature include electron-ion interaction potential (EIIP), atomic number, paired numeric, DNA walk, *Z*-curve representation, molecular mass, and paired nucleotide atomic number [37]. In FM approach, nucleotide sequences are transformed into a series of binary, real, or complex numerical sequences. Examples of FM methods are Voss [38], tetrahedron [39], complex number [40], integer numbers [41], real numbers [42], single Galois indicator [43], quaternary code [44], and left-rotated quaternary code [37].

The Voss, which was named indicator sequence by the proponent, is the first numerical mapping method for DNA sequences [38]. The indicator sequence as defined by Voss is a sequence in which adenine (A), cytosine (C), guanine, (G) and thymine (T) nucleotides are mapped into four binary sequences *x*_*A*_(*k*), *x*_*C*_(*k*), *x*_*G*_(*k*), and *x*_*T*_(*k*), where 1 at position *k* indicates the presence of the base at that position and 0 stands for its absence [38]. The Voss method is very efficient for spectral analysis of nucleotide sequences [36]. It was used in [45] for identification of exons and introns in DNA sequences with appreciable success. The Voss method is applied in this work so as to capture the biological knowledge that are inherent in genomic sequences and to take advantage of the characteristics of the method such as spectral efficiency and 2-dimensional matrix output. With Voss numerical mapping method, there is a good prospect of applying digital image processing (DIP) techniques to obtain descriptors such as HOG and LBP for genomic sequences.

In DIP, an image is a two-dimensional function *g*(*i*, *j*) in which *i* and *j* are spatial coordinates. When *i*, *j* and the amplitude value that is the intensity of *g* are finite, the image is described as a digital image [46]. A *M* × *N* digital grayscale image can be represented in the matrix notation as

(1) $$\begin{array}{c} g\left(i,j\right)=\left[\begin{array}{cccc} g\left(0,0\right) & g\left(0,1\right) & \cdots & g\left(0,N-1\right)\\ g\left(1,0\right) & g\left(1,1\right) & \cdots & g\left(1,N-1\right)\\ \cdots & \cdots & \cdots & \cdots \\ g\left(M-1,0\right) & g\left(M-1,1\right) & \cdots & g\left(M-1,N-1\right) \end{array}\right]. \end{array}$$

Each of the elements of the digital image represented on the right hand side of (1) is called a picture element or pixel. Consequently, with the Voss numerical mapping of genomic nucleotides, the value of zero or one at position *k* of the four sequences *x*_*A*_(*k*), *x*_*C*_(*k*), *x*_*G*_(*k*),* and XT*(*k*) represents the pixel intensity (gray level) at that position. The resulting sequences are concatenated into a 4 × *N* output silhouette matrix similar to (1), where *N* is the total number of bases in a given sequence. The Voss mapping procedure was implemented in this study using the MATLAB R2012a. The Voss mapped sequences for the first ten nucleotides of EGFR, KRAS, and TP53 genes are shown in Tables 3, 4, and 5 for the sake of lucidity.

**Figure 2:**
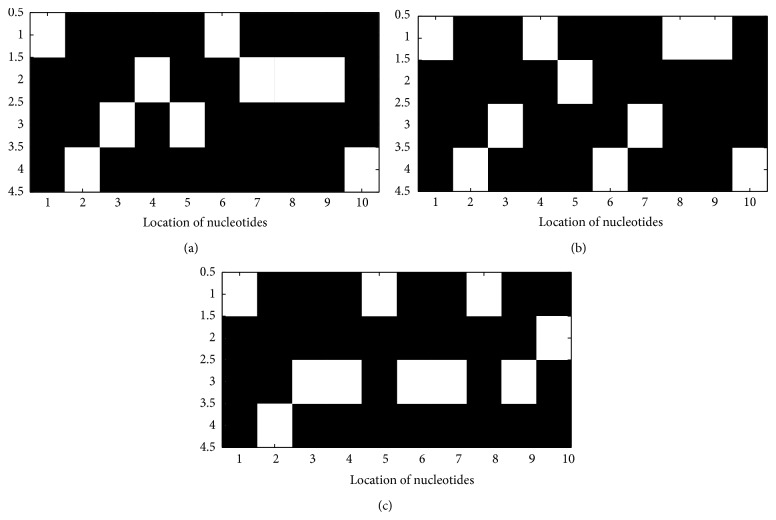
(a) Image of the Voss mapped sequences for the first ten EGFR nucleotides. (b) Image of Voss mapped sequences for the first ten KRAS nucleotides (c) Image of Voss mapped sequences for the first ten TP53 nucleotides.

Image representations for the sequences in Tables 3, 4, and 5 were also obtained using the appropriate functions in MATLAB R2012a and sample outputs are, respectively, shown in Figures 2(a), 2(b), and 2(c). The visual inspection of the figures shows that the images of each of the biomarkers in this study are unique. Hence, we should be able to seek for their unique feature representation to aid efficient lung cancer prediction using machine learning classifiers such as artificial neural networks and support vector machines.

### 2.3. Feature Extraction

The histogram of oriented gradient (HOG) descriptor is explored to extract representative features from the images of the Voss encoded genomic sequences in Section 2.2. The HOG technique, which was developed by Dalal and Triggs [26] for human recognition, is based on the principle that local object appearance and shape in an image can be represented by the distribution of intensity gradients or edge orientations. In order to implement HOG, the image is first divided into cells and histogram of gradient orientations are computed for the pixels within the cells. The resulting histograms are then combined to represent the image descriptor. However, to enhance the performance of the descriptor, local histograms are contrast normalized by computing a measure of intensity across a larger region of the image called a block. The intensity values are then used to normalize all cells within the block, which results in a descriptor that has better invariance to illumination changes and shadowing. There are four primary steps to compute HOG.

The first step involves the computation of the* gradient values,* which can be done by applying the finite difference approximation or derivative masks on the input image. The 1D centered point discrete derivative mask was shown by Dalal and Triggs [26] to be better than Sobel operator and diagonal masks. Using the 1D derivative mask, the input image *I* is filtered in both vertical and horizontal directions with the kernels in

(2) Dx=−101,Dy=10−1T,

where  [·]^*T*^  is a transpose vector. The *x* and *y* derivatives of the silhouette or grayscale image *I* are then obtained using the convolution operation as

(3) $$\begin{array}{c} I_{x}=I*D_{x},\\ I_{y}=I*D_{y}. \end{array}$$

The magnitude and orientation of the gradient of *I* are, respectively, computed using the following:

(4) $$\begin{array}{c} \left|G\right|=\sqrt{I_{x}^{2}+I_{y}^{2}}, \end{array}$$

(5) $$\begin{array}{c} \theta =\arctan\left(\frac{I_{y}}{I_{x}}\right). \end{array}$$

The second step in the computation of HOG is called* orientation binning,* which involves the creation of cell histograms. HOG cells are rectangular (or circular, in some real implementations) and the histogram channels are either unsigned or signed. Signed histogram channels are spread over 0 to 180 degrees, while unsigned channels are spread over 0 to 360 degrees. Using the value in the gradient computation, each pixel within the cell casts a weighted vote for an orientation-based histogram channel. Dalal and Triggs [26] observed that the best experimental result of human recognition was obtained by using unsigned histogram channel and an angular range of 20 degrees. The bin size for this range is therefore 180/20 = 9 histogram channels.

The third step of HOG computation is the creation of* descriptor blocks*. The cell orientation histograms are grouped into larger and spatially connected blocks before they can be normalized. This procedure is carried out so as to account for changes in illumination and contrasts. There are currently two types of geometries for the block, which are rectangular (R-HOG) and circular (C-HOG). The R-HOG is typically a square grid that can be described with the number of cells/block, the number of pixels/cell, and the number of channels/cell histogram. The blocks overlap each other for a magnitude of half size of a block.

The final step in HOG computation is* block normalization*. The normalization factor for a nonnormalized vector (*v*) that contains the histogram in a given block is one of the following norms:

(6) $$\begin{array}{c} \text{L}2\text{-norm:}\;\;f=\frac{v}{\sqrt{{\left\|v\right\|}_{2}^{2}+e^{2}}},\\ \text{L}1\text{-norm:}\;\;f=\frac{v}{{\left\|v\right\|}_{1}+e},\\ \text{L}1\text{-sqrt:}\;\;f=\sqrt{\frac{v}{{\left\|v\right\|}_{1}+e}}, \end{array}$$

where *e* is a constant whose value will not influence the result. Dalal and Triggs [26] observed in their human recognition experiment that L2-norm and L1-sqrt methods produced comparable performance while L1-norm performance is the least. The HOG descriptor is therefore the vector, which contains the normalized cell histograms from all the block regions in the image. In this study, we have applied the unsigned histogram channel and a bin size of 9, similar to the studies in [26, 28] to process the genomic images obtained for both normal and mutation sequences from the Voss mapping procedure discussed in Section 2.2. With this nine-bin size, nine consecutive blocks were then utilized to compute HOG feature vector of size 81 each for all the imaged genomic sequences.

**Figure 3:**
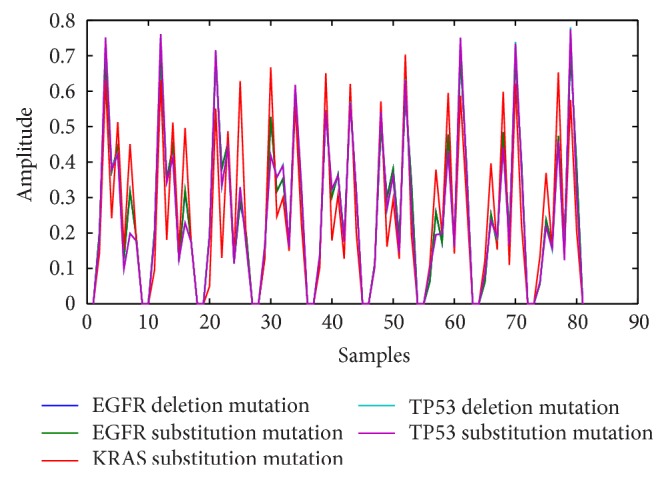
Time domain plot of HOG features for the first samples of EGFR deletion, EGFR substitution, KRAS substitution, TP53 substitution, and TP53 deletion mutations.

The foregoing HOG algorithmic steps were implemented in MATLAB R2012a. Using the results obtained from the code, we plotted the time domain graph of the first samples in each of the classes in our experimental dataset as shown in Figure 3. This graph clearly and visibly shows unique patterns for the different classes of mutations in our training dataset. This is a strong proof of the discriminatory power of HOG descriptor. Our second objective of using Voss mapping to encode and HOG to extract representative genomic features in this study has therefore been realized with the procedures discussed in Section 2.2 and this section. Apart from the first application of HOG descriptor for human recognition by Dalal and Triggs [26], the method has also been used with good results in domains as diverse as activity recognition [28, 47], pedestrian detection [48], and speaker classification [49]. In order to automate the classification of different patterns (mutation classes) captured by the HOG feature vectors in this work, we designed and trained ensemble and nonensemble artificial neural networks and support vector machines.

### 2.4. Classification Models

The classification model for NSCLC in this study classifies an input genomic feature vector into one of six classes in order to predict the presence or absence of specific genomic mutations.  Table 6 shows the different classes in the framework and their numerical representations, which indicate target or expected output from the classification model for each class. Ensemble and nonensemble multilayered perceptron artificial neural network (MLP-ANN) and support vector machine (SVM) are compared in order to make the choice of the most appropriate classification model and to validate our results.

**Figure 4:**
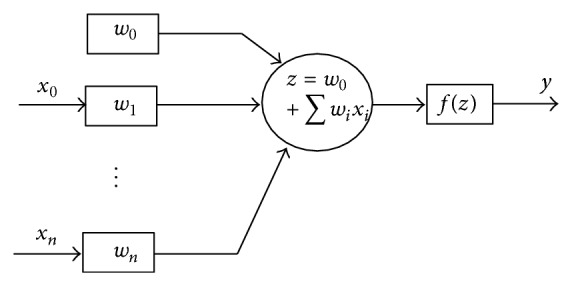
The structure of an artificial neuron with the functional elements.

An artificial neural network (ANN) is a mathematical model that simulates the structure and function of the biological nervous system [50]. It is primarily composed of orderly interconnected artificial neurons. The structure and functional elements of an artificial neuron, which is the building block of all ANN systems, are shown in Figure 4.

As illustrated in Figure 4, an artificial neuron has a set of *n* synapses associated with the inputs (*x*_1_,…, *x*_*n*_) and each input has an associated weight (*w*_*i*_). A signal at input *i* is multiplied by the weight *w*_*i*_, the weighted inputs are added together, and a linear combination of the weighted inputs is obtained. A bias (*w*_0_), which is not associated with any input, is added to the linear combination and a weighted sum *z* is obtained as

(7) $$\begin{array}{c} z=w_{0}+w_{1}x_{1}+\cdots +w_{n}x_{n}. \end{array}$$

Subsequently, a nonlinear activation function *f* is applied to the weighted sum in (7) and this produces an output *y* shown in

(8) $$\begin{array}{c} y=f\left(z\right). \end{array}$$

The flexibility and ability of an artificial neuron to approximate functions to be learned depend on its activation function. Linear and sigmoid functions are some examples of the activation functions frequently used in neural network applications. The linear activation functions are mostly applied in the output layer and it has the form:

(9) $$\begin{array}{c} f\left(z\right)=z. \end{array}$$

The sigmoid activation functions are *S-*shaped and the ones that are mostly used are the logistic and the hyperbolic tangent as represented in (10) and (11), respectively,

(10) $$\begin{array}{c} f\left(z\right)=\frac{1}{1+e^{-az}}, \end{array}$$

(11) $$\begin{array}{c} f\left(z\right)=\frac{e^{z}-e^{-z}}{e^{z}+e^{-z}}. \end{array}$$

One of the most commonly used artificial neural networks is the multilayer perceptron (MLP). The MLP is a nonlinear neural network, which is made up of neurons that are arranged in layers. Typically, MLP is composed of a minimum of three layers, which comprises an input layer, one or more hidden layers, and an output layer [51]. In this study, an MLP topology was designed to learn the extracted genomic features in Section 2.3. The choice of the number of hidden layers is a vital decision to be considered when designing MLP-ANNs. It was established in [52] that a network with one hidden layer can approximate any continuous functions. However, another study [53] has reported that, for large problems, more hidden layers can lead to the training of the network settling for few local minimums and reduction of the network errors. On this basis, we decided to use two hidden layers for the MLP in this study.

The choice of an appropriate activation function for the neurons in the different layers of MLP is very crucial to the performance of the network. The linear activation function is generally used for the input neurons because it transmits the input dataset directly to the next layer with no transformation. The choice of activation function for the output layer neurons is a function of the problem being solved. In our case, which is a multiclass learning problem, we decided to select the hyperbolic tangent sigmoid function for the output layer neurons because it has the capability to handle either continuous values or *n*-class binary classification problems. The hyperbolic tangent sigmoid function is also chosen for the neurons in hidden layers because it is nonlinear and differentiable. Differentiability and nonlinearity are vital requirements for MLP training algorithms [53].

**Figure 5:**
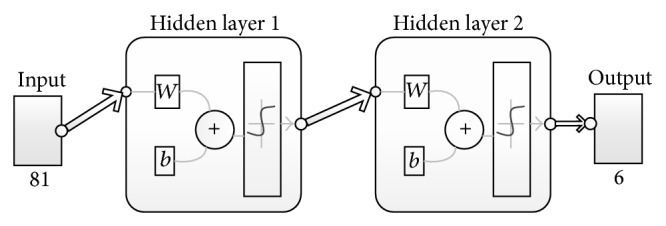
Architecture of the MLP-ANN classifier.

The training dataset for MLP usually consists of a set of patterns (*x*_*p*_, *t*_*p*_) where *p* represents the number of patterns and *x*_*p*_ is the *N*-dimensional input vector. Since each HOG genomic feature vector in this study has 81 elements, *N* is equal to 81 and our *x*_*p*_ is 81-dimensional. Furthermore, *t*_*p*_ is the target output vector for the *p* pattern and because we have six different classes to be classified, we encoded each target output using 6-element binary vector as shown in Table 6. Hence, the MLP architecture in this work contains 81 neurons in the input layer and 6 neurons in the output layer. Based on the foregoing analytical decisions, we designed and configured MLP-ANN in MATLAB R2012a and the resulting network architecture is shown in Figure 5.

MLP neural networks are typically trained with backpropagation (BP) algorithm. BP is an application of the gradient method or other numerical optimization methods to feed-forward ANN so as to minimize the network errors. It is the most popular method for performing supervised learning in ANN research community [54, 55]. There are different variants of the BP algorithm, which include conjugate gradient BP (CGB), scale conjugate gradient (SCG), conjugate gradient BP with Polak-Riebre, conjugate gradient BP with Fletcher-Revees updates, one-secant BP, resilient BP, Levenberg Marquardt (LM), gradient descent, quasi-Newton, and many others [56]. Scaled conjugate gradient backpropagation (SCG-BP) algorithm is a fully automated method, which was designed to avoid the time consuming line search often used in CGB and quasi-Newton BP algorithms [56]. We adopted SCG-BP to train the designed MLP-ANN in this work so as to take advantage of its well acclaimed speed of convergence [30, 57]. The number of neurons in the hidden layer of our MLP was determined experimentally because there is currently no precise rule of thumb for selecting the number of hidden layer neurons [58]. The experimental procedure and the results we obtained are detailed in Section 3.

## 3. Experimental Results and Discussion

The designed MLP-ANN was programmed using the neural network toolbox in MATLAB R2012a. All the experiments reported in this paper were performed on an Intel Core i5-3210M CPU @ 2.50 GHz speed with 6.00 GB RAM and 64-bit Windows 8 operating system. Although training algorithms seek to minimize errors in neural networks, local minimum is often a major problem and one of the important approaches in common use to address this problem is to vary the number of neurons in the hidden layer until an acceptable accuracy is achieved [53]. The first experiment was therefore undertaken to determine the appropriate number of neurons in the hidden layer of our MLP-ANN architecture.

**Figure 6:**
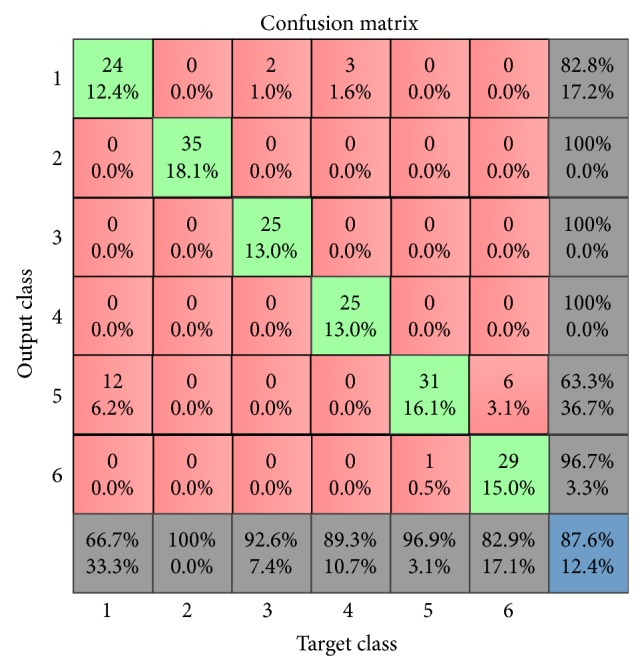
Confusion matrix for the best ANN in Table 7.

**Figure 7:**
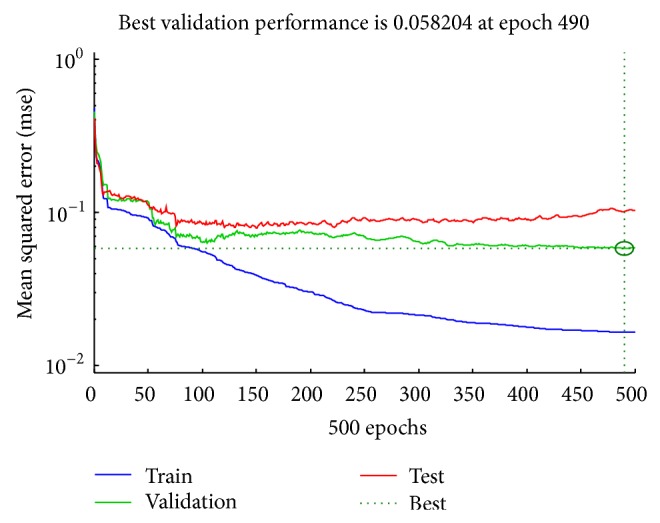
Performance plot for the best ANN in Table 7.

In the first experimental setup, the number of iterations for training the network called epochs in ANN parlance was set to 500. In order to eliminate the incidence of overfitting that may happen, if the number of epochs is either too small or too large, we configured the network to stop the training when the best generalization is reached. This was achieved by partitioning the HOG data into 70% training, 15% validation, and 15% testing subdataset. The HOG training set was used to train the network while the validation set was used to measure the error and the network training stops, when the error starts to increase for the validation dataset. Furthermore, we varied the number of neurons in the hidden layer from 10 in step of 10 to 100 and recorded the mean square errors (MSE) and accuracies (from the confusion matrix plot) for each trial.  Table 7 shows the MSE and accuracies we obtained for the different networks with varying number of neurons in the hidden layer. For the ten different ANN configurations shown in Table 7, the 8th MLP-ANN gave the best accuracy of 87.6%, MSE of 0.0355, and the best validation performance of 0.0584 at 490 epochs. The confusion matrix and the best performance plot of the 8th MLP-ANN are as shown in Figures 6 and 7, respectively. A similar result of 87.2% accuracy was reported by the authors in [30] for a study on the use of SCG-BP for face expression recognition.

From the result of the current experiment, we observed that the performance of the MLP-ANN across each trial did not show any progressive improvement as the number of hidden layer neurons increased. This is illustrated with the lower accuracies of 75.6% in the 9th network and 84.5% in the 10th network. This result is a justification of our decision to experimentally determine the appropriate number of neurons in the hidden layer of the MLP-ANN.

In order to further examine the efficacy of the 8th network, which we adopted based on its performance measures, we tested it with different samples of “seen” and “unseen” HOG genomic features. The “seen” samples are features that were included in the training dataset while the “unseen” samples are features that were not included in the training dataset. The results we obtained are shown in Tables 8 and 9. The result in Table 8 shows that the trained ANN performed brilliantly well when tested on “seen” sample dataset. However, despite the reported accuracy of the 8th MLP-ANN, Table 9 result shows that it performed very poorly on “unseen” dataset. The implication of this result is that the network is overfitted on the training dataset and its generalization capability is very weak. This result is a confirmation of the general criticism in the literature against ANN as a weak and an unstable classifier. In agreement with our result in the current experiment, the authors in [58, 59] also posit that unstable classifiers such as neural network and decision trees always have the problem of high error on test dataset.

However, studies in the literature have affirmed that performance and stability of neural networks can be improved by combining several neural networks, a concept that is known as ensembling [59, 60]. Examples of ensemble methods in the literature include bagging [59], boosting [61], and random forests [62]. In [59], the author stated categorically that bagging ensemble is one of the most effective methods of neural networks combination of learning problems. Consequently, we performed another experiment to determine the effect of bagging ensemble on the performance and stability of the 8th MLP-ANN that we adopted from the first experiment.

In the second experiment, we used the configurations of the 8th MLP-ANN in the first experiment to form the base classifiers of the MLP-ANN bagging ensemble. Bagging is an abbreviation for bootstrap aggregation and it uses a statistical resampling method called bootstrapping to generate multiple training sets. Our training dataset *x*_*p*_ is bootstrapped to form a resampled training set *x*_*p*_^(*s*)^. The resampled dataset is thereafter used to construct a classifier and this procedure is repeated several times to obtain multiple classifiers, which are then combined using an appropriate voting method. The bagging ensemble procedure was implemented in MATLAB R2012a in this study. Using the bagging ensemble implementation, we generated 50 different classifiers and selected the 12 that have accuracies of approximately 95% and above. Selecting the best quality classifiers from multiple ones was also applied to classification trees by the author in [63]. Plurality voting was then applied to the 12 classifiers to obtain the ensemble output. In plurality voting, a prediction is judged as an output, if it comes first in the number of votes that are cast by the base classifiers of the ensemble [64].

In this second experiment, the accuracies and MSEs obtained for the MLP-ANN 12 base classifiers ensemble are shown in Table 10. The result in Table 10 shows an average accuracy of 95.9% and an average MSE of 0.0159 for the MLP-ANN ensemble. This performance is better than the nonensemble MLP-ANN that gave an accuracy of 87.6% and an MSE of about 0.0355 in our previous experiment. In order to further validate the high performance of the MLP-ANN ensemble and examine its level of stability, we tested it with both “seen” and “unseen” HOG genomic samples. The results obtained for the “seen” samples are shown in Table 11 while Table 12 shows the result obtained for “unseen” samples. From Table 11, it can be observed that all the “seen” samples were correctly classified and from Table 12, it can be observed that only one of the “unseen” samples was wrongly classified. However, the results we obtained for nonensemble MLP-ANN in Tables 8 and 9 showed that the nonensemble neural network classifier wrongly classified only one “seen” sample and was able to classify only one “unseen” sample correctly.

In the third experiment, we utilized local binary pattern (LBP) descriptor as a feature extraction algorithm for the Voss-mapped genomics dataset. The goal of this experiment was to experimentally compare the performance of HOG features used in the earlier experiments with LBP features. This is to ascertain the most suitable features for genomic-based lung cancer prediction. LBP is a nonparametric method developed by Ojala et al. [27] for the extraction of local spatial features from images. The theoretical definition of the basic LBP [27, 29] is very simple, which forms the basis of its reputation as a computationally efficient image texture descriptor in the image processing research domain [65, 66]. The MATLAB R2012a implementation of LBP algorithm was applied to the encoded genomic dataset in this study to obtain LBP features for the normal and mutated genomic samples.

**Figure 8:**
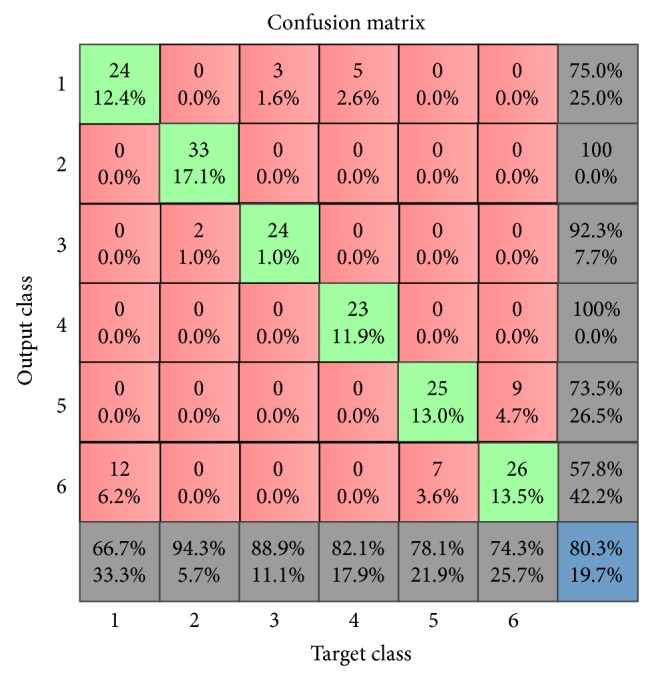
Confusion matrix for the best ANN in Table 13.

Utilizing the same configuration of the nonensemble MLP-ANN in the first experiment, we trained different ANNs with the LBP features by varying the number of hidden layer neurons from 10 to 100 in step of 10. The performance results of different trials are shown in Table 13. As illustrated in the table, the 9th MLP-ANN with 90 neurons in the hidden layer gave the best performance results with an accuracy of 80.3% and MSE of 0.0530. The confusion matrix for this 9th MLP-ANN is shown in Figure 8 and its outputs when tested with “seen” and “unseen” LBP genomic samples are shown in Tables 14 and 15. As illustrated in Table 13, the nonensemble MLP-ANN using LBP features generated poorer accuracy of 80.3% and MSE value of 0.0530 compared to the result in the first experiment in which 87.6% accuracy and MSE of 0.0355 using HOG features were produced. In a similar vein, the outputs in Table 14 show that two instances of “seen” LBP samples were incorrectly classified while, in the first experiment, only one instance of HOG samples was incorrectly classified.  Table 15, however, shows that, similar to the output of the nonensemble MLP-ANN with HOG features in the first experiment, only one instance of “unseen” LBP sample was correctly classified. These statistics apparently show the superiority of HOG genomics features over LBP genomic features and provide evidence that nonensemble MLP-ANN trained with either HOG or LBP features is not suitable for the prediction task in this study.

Furthermore, we followed the bagging ensemble procedure in the second experiment to conduct a fourth experiment. In this fourth experiment, we trained 50 base MLP-ANNs using LBP features and combined the first 12 base MLP-ANNs with the highest accuracies. The results in this fourth experiment gave an average accuracy of 82.4% and an average MSE of 0.0479 (as shown in Table 16). When the MLP-ANN ensemble was tested with “seen” and “unseen” LBP genomic samples, the results we obtained are shown in Tables 17 and 18, respectively. As illustrated in Tables 17 and 18, two samples were misclassified out of eight “seen” LBP samples while two samples were also misclassified out of five “unseen” LBP samples. The second experiment in which MLP-ANN ensemble was trained with HOG genomic samples gave a better result (as shown in Tables 11 and 12) than the results produced by MLP-ANN ensemble trained with LBP genomic samples in the current experiment.

So far, in this section, we have performed four different experiments and compared the performances of HOG and LBP genomic features using both nonensemble MLP-ANN and MLP-ANN ensemble. In order to achieve an elaborate and rigorous comparison of methods for the prediction problem at hand, we undertook further experimenting with both nonensemble support vector machine (SVM) and SVM ensemble classifiers using HOG and LBP genomic features. SVM is a statistical classification method developed by Cortis and Vapnik at Bell Laboratories in 1992 [67]. It has become very popular for supervised learning in fields such as data mining, bioinformatics, and image processing because of its high accuracy and ability to handle data with high dimensionality. Although SVM was first developed for binary classification, it has been improved to cater for multiclass classification by breaking down the multiclass problem into groups of two-class problems [31]. The most common multiclass method used in SVM is one-against-all because it is very efficient and simple [68]. This one-against-all method is adopted for both the nonensemble and ensemble SVMs in the subsequent set of experiments (i.e., experiments 5 and 6) in this study using the implementation in MATLAB R2012a.

It has been established in the literature that SVM can efficiently carry out a nonlinear classification if a good choice of kernel functions is made in its design [32]. The fifth experiment was therefore set up to determine the appropriate kernel function for the SVM classifiers in this study using HOG genomic features and also, to examine the performance of both nonensemble SVM and SVM ensemble on HOG genomic features. For this fifth experiment, we configured the nonensemble SVM with 20-fold cross-validation in which 80% of the HOG genomic samples were used as training set and 20% were implicitly used for validation. In order to determine the kernel function with the best performance metrics for the nonensemble SVM, we tested five different kernel functions, namely, linear, quadratic, polynomial, radial basis function** (**RBF), and multilayer perceptron (MLP) [32]. The performance results we obtained are shown in Table 19.

From Table 19, the polynomial kernel function gave the best accuracy of 86.5% and MSE of 0.0706. The polynomial kernel function was therefore adopted as the kernel function for the SVM classifier in the current experiment.  Table 20 shows the confusion matrix obtained from the properly configured nonensemble SVM, trained with HOG genomic features. The outputs obtained by testing the trained nonensemble SVM in the current experiment using “seen” and “unseen” HOG samples are shown in Tables 21 and 22, respectively. As shown in Tables 21 and 22, all the “seen” samples were correctly classified while two out of the five “unseen” samples were misclassified. This result is an improvement over the result obtained in the first experiment in which nonensemble MLP-ANN misclassified four out of five “unseen” samples. The result in the current experiment further validates the claim in the literature that single SVM has better generalization capability than single neural network [32].

The nonensemble SVM configuration in the current experiment was further utilized to produce SVM ensemble classifier using the bucket-of-models ensemble method. In the bucket-of-models ensemble, the classification result of the best model in the bucket for the problem being solved is often selected [69]. The HOG genomic features were used to train 50 base SVMs to obtain an SVM ensemble classifier and the best model in the ensemble gave an accuracy of 91.9% with MSE of 0.0692. The outputs obtained when the SVM ensemble was tested with “seen” and “unseen” HOG genomic samples are shown in Tables 23 and 24. The results in Tables 23 and 24 show that all the “seen” samples were correctly classified while two out of the five “unseen” samples were misclassified. These results imply that SVM ensemble does not have a radical improvement over the nonensemble SVM. However, comparing the current result with the result in the second experiment, the MLP-ANN ensemble performs better than SVM ensemble using the HOG genomic datasets to train the two ensemble classifiers.

The sixth experiment, which is the last in this study, was targeted at examining the performance of nonensemble and ensemble SVM on LBP genomic features. The first step we took to achieve this objective was to test the five different kernel functions so as to determine the best one for a nonensemble SVM using LBP genomic features. Using 20-fold cross-validation in which 80% of the LBP samples were used for training and 20% utilized for validation, the performance result we obtained is shown in Table 25. The table shows that the polynomial kernel gave the best performance with an accuracy of 75.7% and MSE of 0.0785. The confusion matrix obtained from the nonensemble SVM trained with LBP features in the current experiment is shown in Table 26 and the outputs when tested with “seen” and “unseen” LBP features are shown in Tables 25 and 26. The results obtained in the current experiment are not as good as what we obtained in the fifth experiment in which polynomial kernel function gave an accuracy of 86.5% and MSE of 0.0691 for a nonensemble SVM trained with HOG genomic features. Moreover, in Tables 27 and 28, three out of eight samples were misclassified in the “seen” LBP samples while one out of five samples was misclassified in the “unseen” LBP samples. On a general basis, the result in the current experiment is not as good as the result we obtained in the fifth experiment in which nonensemble SVM was trained with HOG genomic features.

In furtherance of the realization of the objectives of the current experiment, we utilized the same ensemble strategy in the fifth experiment to train 50 base SVMs using LBP features to obtain SVM ensemble. The model with the best performance in the SVM ensemble gave an accuracy of 75.7% and MSE of 0.0743. The outputs we obtained from the SVM ensemble on both “seen” and “unseen” LBP genomic samples are shown in Tables 29 and 30. As illustrated in Tables 29 and 30, three out of eight “seen” LBP samples were misclassified while one out of five “unseen” samples was misclassified. The general results obtained in the fifth experiment from SVM ensemble trained with HOG genomic samples are better than the results of the SVM ensemble with LBP genomic samples in the current experiment.

So far, in this section, we have meticulously experimented with different classifiers and features extraction algorithms so as to arrive at a robust decision on the choice of models for the lung cancer prediction framework being proposed in this study. Hence, the summary of the accuracies and MSEs of the different combinations of classifiers and feature extraction methods in the foregoing experiments are shown in Table 31.

As shown in Table 31, the result in the second experiment in which an accuracy of 95.9 and MSE of 0.0159 was obtained provides a strong validation of the ability of MLP-ANN ensemble to give high performance and high stability on the test dataset of HOG genomic features. Based on this level of performance compared to the other models shown in Table 31, the MLP-ANN ensemble is recommended as the classifier and HOG as the feature descriptor in the NSCLC prediction framework being proposed in this study. The result, we obtained in this work, is also in conformity with the study on lung cancer cell identification based on artificial neural network ensemble [70], where the images of the specimen of needle biopsies were obtained from patients as the dataset. The single ANN in [70] gave a poor average error of 45.5% and neural network ensemble-based detection (NED) system proposed in the study gave an average error of 11.6% as reported [70].

## 4. Conclusion

In this paper, we propose artificial neural network ensemble with histogram of oriented gradient genomic features for lung cancer prediction. The proposed framework has several advantages, which include automated prediction using artificial neural network ensemble, multiple biomarkers for lung cancer on a single platform, compliance with NGS genomic-based technology, and high prediction accuracy. The performance comparison of the proposed framework with support vector machine and local binary pattern is valuable for decision makers to consider tradeoffs in method accuracy versus method complexity. In the future, we hope to incorporate more biomarkers on the proposed platform and carry out further intensive comparative studies using other state-of-the-art machine learning algorithms and features extraction methods.

## Figures and Tables

**Table 1:** Normal gene characteristics.

S/N	Gene symbol	Number of nucleotides	CCDS ID
1	EGFR	3633	CCDS 5514.1
2	KRAS	567	CCDS 8702.1
3	TP53	1182	CCDS 11118.1

**Table 2:** Mutation class characteristics.

S/N	Mutation class	Number of acquired samples	Number of unique samples
1	EGFR deletion	2640	35
2	EGFR substitution	975	27
3	KRAS substitution	2472	28
4	TP53 deletion	42	32
5	TP53 substitution	277	35
	Total	**6,406**	**157**

**Table 3:** Voss mapping of the first ten EGFR gene nucleotides.

DNA sequence	A	T	G	C	G	A	C	C	C	T…
*x*_*A*_(*k*):	1	0	0	0	0	1	0	0	0	0
*x*_*C*_(*k*):	0	0	0	1	0	0	1	1	1	0
*x*_*G*_(*k*):	0	0	1	0	1	0	0	0	0	0
*x*_*T*_(*k*):	0	1	0	0	0	0	0	0	0	1

**Table 4:** Voss mapping of the first ten KRAS gene nucleotides.

DNA sequence	A	T	G	A	C	T	G	A	A	T…
*x*_*A*_(*k*):	1	0	0	1	0	0	0	1	1	0
*x*_*C*_(*k*):	0	0	0	0	1	0	0	0	0	0
*x*_*G*_(*k*):	0	0	1	0	0	0	1	0	0	0
*x*_*T*_(*k*):	0	1	0	0	0	1	0	0	0	1

**Table 5:** Voss mapping of the first ten TP53 gene nucleotides.

DNA sequence	A	T	G	G	A	G	G	A	G	C…
*x*_*A*_(*k*):	1	0	0	0	1	0	0	1	0	0
*x*_*C*_(*k*):	0	0	0	0	0	0	0	0	0	1
*x*_*G*_(*k*):	0	0	1	1	0	1	1	0	1	0
*x*_*T*_(*k*):	0	1	0	0	0	0	0	0	0	0

**Table 6:** Numerical representation which indicates the target output of different classes.

S/N	Class	MLP-ANN target output	SVM target output
1	Normal (EGFR/KRAS/TP53)	$\begin{array}{ccc} 1 & 0 & \begin{array}{ccc} 0 & 0 & \begin{array}{cc} 0 & 0 \end{array} \end{array} \end{array}$	1
2	EGFR deletion	$\begin{array}{ccc} 0 & 1 & \begin{array}{ccc} 0 & 0 & \begin{array}{cc} 0 & 0 \end{array} \end{array} \end{array}$	2
3	EGFR substitution	$\begin{array}{ccc} 0 & 0 & \begin{array}{ccc} 1 & 0 & \begin{array}{cc} 0 & 0 \end{array} \end{array} \end{array}$	3
4	KRAS substitution	$\begin{array}{ccc} 0 & 0 & \begin{array}{ccc} 0 & 1 & \begin{array}{cc} 0 & 0 \end{array} \end{array} \end{array}$	4
5	TP53 deletion	$\begin{array}{ccc} 0 & 0 & \begin{array}{ccc} 0 & 0 & \begin{array}{cc} 1 & 0 \end{array} \end{array} \end{array}$	5
6	TP53 substitution	$\begin{array}{ccc} 0 & 0 & \begin{array}{ccc} 0 & 0 & \begin{array}{cc} 0 & 1 \end{array} \end{array} \end{array}$	6

**Table 7:** Nonensemble MLP-ANN experimentation result with varying number of hidden layer neurons using HOG features.

MLP-ANN	Hidden layer neurons	MSE	Accuracy (%)
1	10	0.0605	75.1
2	20	0.0867	61.1
3	30	0.0380	71.5
4	40	0.0581	74.6
5	50	0.0439	80.3
6	60	0.0516	79.3
7	70	0.0614	78.8
**8**	**80**	**0.0355**	**87.6**
9	90	0.0604	75.6
10	100	0.0403	84.5

**Table 8:** Output of the nonensemble MLP-ANN on “seen” HOG genomics samples.

S/N	Class name	Actual output	Target output	Remark
1	Normal			
	EGFR	$\begin{array}{ccc} 1 & 0 & \begin{array}{ccc} 0 & 0 & \begin{array}{cc} 0 & 0 \end{array} \end{array} \end{array}$	$\begin{array}{ccc} 1 & 0 & \begin{array}{ccc} 0 & 0 & \begin{array}{cc} 0 & 0 \end{array} \end{array} \end{array}$	Correct prediction
	KRAS	$\begin{array}{ccc} 1 & 0 & \begin{array}{ccc} 0 & 0 & \begin{array}{cc} 0 & 0 \end{array} \end{array} \end{array}$	$\begin{array}{ccc} 1 & 0 & \begin{array}{ccc} 0 & 0 & \begin{array}{cc} 0 & 0 \end{array} \end{array} \end{array}$	Correct prediction
	TP53	$\begin{array}{ccc} 0 & 0 & \begin{array}{ccc} 0 & 0 & \begin{array}{cc} 1 & 0 \end{array} \end{array} \end{array}$	$\begin{array}{ccc} 1 & 0 & \begin{array}{ccc} 0 & 0 & \begin{array}{cc} 0 & 0 \end{array} \end{array} \end{array}$	Incorrect prediction
2	EGFR deletion	$\begin{array}{ccc} 0 & 1 & \begin{array}{ccc} 0 & 0 & \begin{array}{cc} 0 & 0 \end{array} \end{array} \end{array}$	$\begin{array}{ccc} 0 & 1 & \begin{array}{ccc} 0 & 0 & \begin{array}{cc} 0 & 0 \end{array} \end{array} \end{array}$	Correct prediction
3	EGFR substitution	$\begin{array}{ccc} 0 & 0 & \begin{array}{ccc} 1 & 0 & \begin{array}{cc} 0 & 0 \end{array} \end{array} \end{array}$	$\begin{array}{ccc} 0 & 0 & \begin{array}{ccc} 1 & 0 & \begin{array}{cc} 0 & 0 \end{array} \end{array} \end{array}$	Correct prediction
4	KRAS substitution	$\begin{array}{ccc} 0 & 0 & \begin{array}{ccc} 0 & 1 & \begin{array}{cc} 0 & 0 \end{array} \end{array} \end{array}$	$\begin{array}{ccc} 0 & 0 & \begin{array}{ccc} 0 & 1 & \begin{array}{cc} 0 & 0 \end{array} \end{array} \end{array}$	Correct prediction
5	TP53 deletion	$\begin{array}{ccc} 0 & 0 & \begin{array}{ccc} 0 & 0 & \begin{array}{cc} 1 & 0 \end{array} \end{array} \end{array}$	$\begin{array}{ccc} 0 & 0 & \begin{array}{ccc} 0 & 0 & \begin{array}{cc} 1 & 0 \end{array} \end{array} \end{array}$	Correct prediction
6	TP53 substitution	$\begin{array}{ccc} 0 & 0 & \begin{array}{ccc} 0 & 0 & \begin{array}{cc} 0 & 1 \end{array} \end{array} \end{array}$	$\begin{array}{ccc} 0 & 0 & \begin{array}{ccc} 0 & 0 & \begin{array}{cc} 0 & 1 \end{array} \end{array} \end{array}$	Correct prediction

**Table 9:** Output of the nonensemble MLP-ANN on “unseen” HOG genomic samples.

S/N	Class names	Actual output	Target output	Remark
1	EGFR deletion	$\begin{array}{ccc} 0 & 1 & \begin{array}{ccc} 0 & 0 & \begin{array}{cc} 0 & 0 \end{array} \end{array} \end{array}$	$\begin{array}{ccc} 0 & 1 & \begin{array}{ccc} 0 & 0 & \begin{array}{cc} 0 & 0 \end{array} \end{array} \end{array}$	Correct prediction
2	EGFR substitution	$\begin{array}{ccc} 1 & 0 & \begin{array}{ccc} 1 & 0 & \begin{array}{cc} 0 & 0 \end{array} \end{array} \end{array}$	$\begin{array}{ccc} 0 & 0 & \begin{array}{ccc} 1 & 0 & \begin{array}{cc} 0 & 0 \end{array} \end{array} \end{array}$	Incorrect prediction
3	KRAS substitution	$\begin{array}{ccc} 1 & 0 & \begin{array}{ccc} 0 & 0 & \begin{array}{cc} 0 & 0 \end{array} \end{array} \end{array}$	$\begin{array}{ccc} 0 & 0 & \begin{array}{ccc} 0 & 1 & \begin{array}{cc} 0 & 0 \end{array} \end{array} \end{array}$	Incorrect prediction
4	TP53 deletion	$\begin{array}{ccc} 0 & 0 & \begin{array}{ccc} 0 & 0 & \begin{array}{cc} 0 & 1 \end{array} \end{array} \end{array}$	$\begin{array}{ccc} 0 & 0 & \begin{array}{ccc} 0 & 0 & \begin{array}{cc} 1 & 0 \end{array} \end{array} \end{array}$	Incorrect prediction
5	TP53 substitution	$\begin{array}{ccc} 0 & 0 & \begin{array}{ccc} 0 & 0 & \begin{array}{cc} 1 & 0 \end{array} \end{array} \end{array}$	$\begin{array}{ccc} 0 & 0 & \begin{array}{ccc} 0 & 0 & \begin{array}{cc} 0 & 1 \end{array} \end{array} \end{array}$	Incorrect prediction

**Table 10:** Result of base classifiers in the MLP-ANN ensemble using HOG genomic samples.

Base MLP-ANN	MSE	Accuracy (%)
1	0.0170	95.9
2	0.0118	97.9
3	0.0232	94.8
4	0.0107	97.9
5	0.0088	97.9
6	0.0193	94.8
7	0.0215	95.3
8	0.0206	94.8
9	0.0157	95.9
10	0.0197	94.8
11	0.0165	95.9
12	0.0222	94.8
Total	**0.1905**	**1150.7**
Average	**0.0159**	**95.9**

**Table 11:** Output of the MLP-ANN ensemble on “seen” HOG genomic samples.

S/N	Class name	Actual output	Target output	Remark
1	Normal			
	EGFR	$\begin{array}{ccc} 1 & 0 & \begin{array}{ccc} 0 & 0 & \begin{array}{cc} 0 & 0 \end{array} \end{array} \end{array}$	$\begin{array}{ccc} 1 & 0 & \begin{array}{ccc} 0 & 0 & \begin{array}{cc} 0 & 0 \end{array} \end{array} \end{array}$	Correct prediction
	KRAS	$\begin{array}{ccc} 1 & 0 & \begin{array}{ccc} 0 & 0 & \begin{array}{cc} 0 & 0 \end{array} \end{array} \end{array}$	$\begin{array}{ccc} 1 & 0 & \begin{array}{ccc} 0 & 0 & \begin{array}{cc} 0 & 0 \end{array} \end{array} \end{array}$	Correct prediction
	TP53	$\begin{array}{ccc} 1 & 0 & \begin{array}{ccc} 0 & 0 & \begin{array}{cc} 0 & 0 \end{array} \end{array} \end{array}$	$\begin{array}{ccc} 1 & 0 & \begin{array}{ccc} 0 & 0 & \begin{array}{cc} 0 & 0 \end{array} \end{array} \end{array}$	Correct prediction
2	EGFR deletion	$\begin{array}{ccc} 0 & 1 & \begin{array}{ccc} 0 & 0 & \begin{array}{cc} 0 & 0 \end{array} \end{array} \end{array}$	$\begin{array}{ccc} 0 & 1 & \begin{array}{ccc} 0 & 0 & \begin{array}{cc} 0 & 0 \end{array} \end{array} \end{array}$	Correct prediction
3	EGFR substitution	$\begin{array}{ccc} 0 & 0 & \begin{array}{ccc} 1 & 0 & \begin{array}{cc} 0 & 0 \end{array} \end{array} \end{array}$	$\begin{array}{ccc} 0 & 0 & \begin{array}{ccc} 1 & 0 & \begin{array}{cc} 0 & 0 \end{array} \end{array} \end{array}$	Correct prediction
4	KRAS substitution	$\begin{array}{ccc} 0 & 0 & \begin{array}{ccc} 0 & 1 & \begin{array}{cc} 0 & 0 \end{array} \end{array} \end{array}$	$\begin{array}{ccc} 0 & 0 & \begin{array}{ccc} 0 & 1 & \begin{array}{cc} 0 & 0 \end{array} \end{array} \end{array}$	Correct prediction
5	TP53 deletion	$\begin{array}{ccc} 0 & 0 & \begin{array}{ccc} 0 & 0 & \begin{array}{cc} 1 & 0 \end{array} \end{array} \end{array}$	$\begin{array}{ccc} 0 & 0 & \begin{array}{ccc} 0 & 0 & \begin{array}{cc} 1 & 0 \end{array} \end{array} \end{array}$	Correct prediction
6	TP53 substitution	$\begin{array}{ccc} 0 & 0 & \begin{array}{ccc} 0 & 0 & \begin{array}{cc} 0 & 1 \end{array} \end{array} \end{array}$	$\begin{array}{ccc} 0 & 0 & \begin{array}{ccc} 0 & 0 & \begin{array}{cc} 0 & 1 \end{array} \end{array} \end{array}$	Correct prediction

**Table 12:** Output of the MLP-ANN ensemble on “unseen” HOG genomic samples.

S/N	Output class	Actual output	Target output	Remark
1	EGFR deletion	$\begin{array}{ccc} 0 & 1 & \begin{array}{ccc} 0 & 0 & \begin{array}{cc} 0 & 0 \end{array} \end{array} \end{array}$	$\begin{array}{ccc} 0 & 1 & \begin{array}{ccc} 0 & 0 & \begin{array}{cc} 0 & 0 \end{array} \end{array} \end{array}$	Correct prediction
2	EGFR substitution	$\begin{array}{ccc} 0 & 0 & \begin{array}{ccc} 1 & 0 & \begin{array}{cc} 0 & 0 \end{array} \end{array} \end{array}$	$\begin{array}{ccc} 0 & 0 & \begin{array}{ccc} 1 & 0 & \begin{array}{cc} 0 & 0 \end{array} \end{array} \end{array}$	Correct prediction
3	KRAS substitution	$\begin{array}{ccc} 0 & 0 & \begin{array}{ccc} 0 & 1 & \begin{array}{cc} 0 & 0 \end{array} \end{array} \end{array}$	$\begin{array}{ccc} 0 & 0 & \begin{array}{ccc} 0 & 1 & \begin{array}{cc} 0 & 0 \end{array} \end{array} \end{array}$	Correct prediction
4	TP53 deletion	$\begin{array}{ccc} 0 & 0 & \begin{array}{ccc} 0 & 0 & \begin{array}{cc} 1 & 0 \end{array} \end{array} \end{array}$	$\begin{array}{ccc} 0 & 0 & \begin{array}{ccc} 0 & 0 & \begin{array}{cc} 1 & 0 \end{array} \end{array} \end{array}$	Correct prediction
5	TP53 substitution	$\begin{array}{ccc} 1 & 0 & \begin{array}{ccc} 0 & 0 & \begin{array}{cc} 0 & 0 \end{array} \end{array} \end{array}$	$\begin{array}{ccc} 0 & 0 & \begin{array}{ccc} 0 & 0 & \begin{array}{cc} 0 & 1 \end{array} \end{array} \end{array}$	Incorrect prediction

**Table 13:** Nonensemble MLP-ANN result with varying number of hidden layer neurons using LBP genomic samples.

MLP-ANN	Hidden layer neurons	MSE	Accuracy (%)
1	10	0.0667	74.1
2	20	0.0628	71.5
3	30	0.0693	74.6
4	40	0.0632	71.5
5	50	0.0623	76.2
6	60	0.0585	74.1
7	70	0.0616	71.5
8	80	0.0504	77.7
**9**	**90**	**0.0530**	**80.3**
10	100	0.0555	78.2

**Table 14:** Output of the nonensemble MLP-ANN on “seen” LBP genomic samples.

S/N	Class name	Actual output	Target output	Remark
1	Normal			
	EGFR	$\begin{array}{ccc} 1 & 0 & \begin{array}{ccc} 0 & 0 & \begin{array}{cc} 0 & 0 \end{array} \end{array} \end{array}$	$\begin{array}{ccc} 1 & 0 & \begin{array}{ccc} 0 & 0 & \begin{array}{cc} 0 & 0 \end{array} \end{array} \end{array}$	Correct prediction
	KRAS	$\begin{array}{ccc} 1 & 0 & \begin{array}{ccc} 0 & 0 & \begin{array}{cc} 0 & 0 \end{array} \end{array} \end{array}$	$\begin{array}{ccc} 1 & 0 & \begin{array}{ccc} 0 & 0 & \begin{array}{cc} 0 & 0 \end{array} \end{array} \end{array}$	Correct prediction
	TP53	$\begin{array}{ccc} 0 & 0 & \begin{array}{ccc} 0 & 0 & \begin{array}{cc} 0 & 0 \end{array} \end{array} \end{array}$	$\begin{array}{ccc} 1 & 0 & \begin{array}{ccc} 0 & 0 & \begin{array}{cc} 0 & 0 \end{array} \end{array} \end{array}$	Incorrect prediction
2	EGFR deletion	$\begin{array}{ccc} 0 & 1 & \begin{array}{ccc} 0 & 0 & \begin{array}{cc} 0 & 0 \end{array} \end{array} \end{array}$	$\begin{array}{ccc} 0 & 1 & \begin{array}{ccc} 0 & 0 & \begin{array}{cc} 0 & 0 \end{array} \end{array} \end{array}$	Correct prediction
3	EGFR substitution	$\begin{array}{ccc} 0 & 0 & \begin{array}{ccc} 1 & 0 & \begin{array}{cc} 0 & 0 \end{array} \end{array} \end{array}$	$\begin{array}{ccc} 0 & 0 & \begin{array}{ccc} 1 & 0 & \begin{array}{cc} 0 & 0 \end{array} \end{array} \end{array}$	Correct prediction
4	KRAS substitution	$\begin{array}{ccc} 1 & 0 & \begin{array}{ccc} 0 & 0 & \begin{array}{cc} 0 & 0 \end{array} \end{array} \end{array}$	$\begin{array}{ccc} 0 & 0 & \begin{array}{ccc} 0 & 1 & \begin{array}{cc} 0 & 0 \end{array} \end{array} \end{array}$	Incorrect prediction
5	TP53 deletion	$\begin{array}{ccc} 0 & 0 & \begin{array}{ccc} 0 & 0 & \begin{array}{cc} 1 & 0 \end{array} \end{array} \end{array}$	$\begin{array}{ccc} 0 & 0 & \begin{array}{ccc} 0 & 0 & \begin{array}{cc} 1 & 0 \end{array} \end{array} \end{array}$	Correct prediction
6	TP53 substitution	$\begin{array}{ccc} 0 & 0 & \begin{array}{ccc} 0 & 0 & \begin{array}{cc} 0 & 1 \end{array} \end{array} \end{array}$	$\begin{array}{ccc} 0 & 0 & \begin{array}{ccc} 0 & 0 & \begin{array}{cc} 0 & 1 \end{array} \end{array} \end{array}$	Correct prediction

**Table 15:** Output of the nonensemble MLP-ANN on “unseen” LBP genomic samples.

S/N	Class names	Actual output	Target output	Remark
1	EGFR deletion	$\begin{array}{ccc} 0 & 1 & \begin{array}{ccc} 0 & 0 & \begin{array}{cc} 0 & 0 \end{array} \end{array} \end{array}$	$\begin{array}{ccc} 0 & 1 & \begin{array}{ccc} 0 & 0 & \begin{array}{cc} 0 & 0 \end{array} \end{array} \end{array}$	Correct prediction
2	EGFR substitution	$\begin{array}{ccc} 1 & 0 & \begin{array}{ccc} 0 & 0 & \begin{array}{cc} 0 & 0 \end{array} \end{array} \end{array}$	$\begin{array}{ccc} 0 & 0 & \begin{array}{ccc} 1 & 0 & \begin{array}{cc} 0 & 0 \end{array} \end{array} \end{array}$	Incorrect prediction
3	KRAS substitution	$\begin{array}{ccc} 1 & 0 & \begin{array}{ccc} 0 & 0 & \begin{array}{cc} 0 & 0 \end{array} \end{array} \end{array}$	$\begin{array}{ccc} 0 & 0 & \begin{array}{ccc} 0 & 1 & \begin{array}{cc} 0 & 0 \end{array} \end{array} \end{array}$	Incorrect prediction
4	TP53 deletion	$\begin{array}{ccc} 0 & 0 & \begin{array}{ccc} 0 & 0 & \begin{array}{cc} 0 & 1 \end{array} \end{array} \end{array}$	$\begin{array}{ccc} 0 & 0 & \begin{array}{ccc} 0 & 0 & \begin{array}{cc} 1 & 0 \end{array} \end{array} \end{array}$	Incorrect prediction
5	TP53 substitution	$\begin{array}{ccc} 0 & 0 & \begin{array}{ccc} 0 & 0 & \begin{array}{cc} 0 & 0 \end{array} \end{array} \end{array}$	$\begin{array}{ccc} 0 & 0 & \begin{array}{ccc} 0 & 0 & \begin{array}{cc} 0 & 1 \end{array} \end{array} \end{array}$	Incorrect prediction

**Table 16:** Result of base classifiers in MLP-ANN ensemble using LBP genomic samples.

Base MLP-ANN	MSE	Accuracy (%)
1	0.0479	87.0
2	0.0464	81.3
3	0.0513	81.3
4	0.0450	82.9
5	0.0446	79.8
6	0.0496	80.3
7	0.0462	81.3
8	0.0522	80.8
9	0.0449	82.9
10	0.0501	79.8
11	0.0491	83.9
12	0.0476	87.6
Total	**0.5749**	**988.9**
Average	**0.0479**	**82.4**

**Table 17:** Output of the MLP-ANN ensemble on “seen” LBP genomic samples.

S/N	Class name	Actual output	Target output	Remark
1	Normal			
	EGFR	$\begin{array}{ccc} 1 & 0 & \begin{array}{ccc} 0 & 0 & \begin{array}{cc} 0 & 0 \end{array} \end{array} \end{array}$	$\begin{array}{ccc} 1 & 0 & \begin{array}{ccc} 0 & 0 & \begin{array}{cc} 0 & 0 \end{array} \end{array} \end{array}$	Correct prediction
	KRAS	$\begin{array}{ccc} 0 & 0 & \begin{array}{ccc} 0 & 1 & \begin{array}{cc} 0 & 0 \end{array} \end{array} \end{array}$	$\begin{array}{ccc} 1 & 0 & \begin{array}{ccc} 0 & 0 & \begin{array}{cc} 0 & 0 \end{array} \end{array} \end{array}$	Incorrect prediction
	TP53	$\begin{array}{ccc} 0 & 0 & \begin{array}{ccc} 0 & 0 & \begin{array}{cc} 0 & 0 \end{array} \end{array} \end{array}$	$\begin{array}{ccc} 1 & 0 & \begin{array}{ccc} 0 & 0 & \begin{array}{cc} 0 & 0 \end{array} \end{array} \end{array}$	Incorrect prediction
2	EGFR deletion	$\begin{array}{ccc} 0 & 1 & \begin{array}{ccc} 0 & 0 & \begin{array}{cc} 0 & 0 \end{array} \end{array} \end{array}$	$\begin{array}{ccc} 0 & 1 & \begin{array}{ccc} 0 & 0 & \begin{array}{cc} 0 & 0 \end{array} \end{array} \end{array}$	Correct prediction
3	EGFR substitution	$\begin{array}{ccc} 0 & 0 & \begin{array}{ccc} 1 & 0 & \begin{array}{cc} 0 & 0 \end{array} \end{array} \end{array}$	$\begin{array}{ccc} 0 & 0 & \begin{array}{ccc} 1 & 0 & \begin{array}{cc} 0 & 0 \end{array} \end{array} \end{array}$	Correct prediction
4	KRAS substitution	$\begin{array}{ccc} 0 & 0 & \begin{array}{ccc} 0 & 1 & \begin{array}{cc} 0 & 0 \end{array} \end{array} \end{array}$	$\begin{array}{ccc} 0 & 0 & \begin{array}{ccc} 0 & 1 & \begin{array}{cc} 0 & 0 \end{array} \end{array} \end{array}$	Correct prediction
5	TP53 deletion	$\begin{array}{ccc} 0 & 0 & \begin{array}{ccc} 0 & 0 & \begin{array}{cc} 1 & 0 \end{array} \end{array} \end{array}$	$\begin{array}{ccc} 0 & 0 & \begin{array}{ccc} 0 & 0 & \begin{array}{cc} 1 & 0 \end{array} \end{array} \end{array}$	Correct prediction
6	TP53 substitution	$\begin{array}{ccc} 0 & 0 & \begin{array}{ccc} 0 & 0 & \begin{array}{cc} 0 & 1 \end{array} \end{array} \end{array}$	$\begin{array}{ccc} 0 & 0 & \begin{array}{ccc} 0 & 0 & \begin{array}{cc} 0 & 1 \end{array} \end{array} \end{array}$	Correct prediction

**Table 18:** Output of the MLP-ANN ensemble on “unseen” LBP genomics samples.

S/N	Output class	Actual output	Target output	Remark
1	EGFR deletion	$\begin{array}{ccc} 0 & 1 & \begin{array}{ccc} 0 & 0 & \begin{array}{cc} 0 & 0 \end{array} \end{array} \end{array}$	$\begin{array}{ccc} 0 & 1 & \begin{array}{ccc} 0 & 0 & \begin{array}{cc} 0 & 0 \end{array} \end{array} \end{array}$	Correct prediction
2	EGFR substitution	$\begin{array}{ccc} 1 & 0 & \begin{array}{ccc} 0 & 0 & \begin{array}{cc} 0 & 0 \end{array} \end{array} \end{array}$	$\begin{array}{ccc} 0 & 0 & \begin{array}{ccc} 1 & 0 & \begin{array}{cc} 0 & 0 \end{array} \end{array} \end{array}$	Incorrect prediction
3	KRAS substitution	$\begin{array}{ccc} 0 & 0 & \begin{array}{ccc} 0 & 1 & \begin{array}{cc} 0 & 0 \end{array} \end{array} \end{array}$	$\begin{array}{ccc} 0 & 0 & \begin{array}{ccc} 0 & 1 & \begin{array}{cc} 0 & 0 \end{array} \end{array} \end{array}$	Correct prediction
4	TP53 deletion	$\begin{array}{ccc} 0 & 0 & \begin{array}{ccc} 0 & 0 & \begin{array}{cc} 1 & 0 \end{array} \end{array} \end{array}$	$\begin{array}{ccc} 0 & 0 & \begin{array}{ccc} 0 & 0 & \begin{array}{cc} 1 & 0 \end{array} \end{array} \end{array}$	Correct prediction
5	TP53 substitution	$\begin{array}{ccc} 0 & 0 & \begin{array}{ccc} 0 & 0 & \begin{array}{cc} 1 & 0 \end{array} \end{array} \end{array}$	$\begin{array}{ccc} 0 & 0 & \begin{array}{ccc} 0 & 0 & \begin{array}{cc} 0 & 1 \end{array} \end{array} \end{array}$	Incorrect prediction

**Table 19:** Nonensemble SVM experimentation result with varying kernel functions using HOG genomic samples.

S/N	Kernel function	MSE	Accuracy (%)
1	Linear	0.0825	67.6
2	Quadratic	0.0735	81.1
**3**	**Polynomial**	**0.0706**	**86.5**
4	RBF	0.0709	78.4
5	MLP	0.0926	40.5

**Table 20:** Confusion matrix of the nonensemble SVM with polynomial kernel function using HOG genomic samples.

a	b	c	d	e	f	Classified as
7	0	0	0	0	0	a = normal
0	7	0	0	0	0	b = EGFR deletion
1	0	4	0	0	0	c = EGFR substitution
1	0	0	4	0	0	d = KRAS substitution
1	0	0	0	5	0	e = TP53 deletion
2	0	0	0	0	5	f = TP53 substitution

**Table 21:** Output of the nonensemble SVM on “seen” HOG genomic samples.

S/N	Class name	Actual output	Target output	Remark
1	Normal			
	EGFR	1	1	Correct prediction
	KRAS	1	1	Correct prediction
	TP53	1	1	Correct prediction
2	EGFR deletion	2	2	Correct prediction
3	EGFR substitution	3	3	Correct prediction
4	KRAS substitution	4	4	Correct prediction
5	TP53 deletion	5	5	Correct prediction
6	TP53 substitution	6	6	Correct prediction

**Table 22:** Output of the nonensemble SVM on “unseen” HOG genomic samples.

S/N	Output class	Actual output	Target output	Remark
1	EGFR deletion	2	2	Correct prediction
2	EGFR substitution	1	3	Incorrect prediction
3	KRAS substitution	1	4	Incorrect prediction
4	TP53 deletion	5	5	Correct prediction
5	TP53 substitution	6	6	Correct prediction

**Table 23:** Output of the SVM ensemble on “seen” HOG genomic samples.

S/N	Class name	Actual output	Target output	Remark
1	Normal			
	EGFR	1	1	Correct prediction
	KRAS	1	1	Correct prediction
	TP53	1	1	Correct prediction
2	EGFR deletion	2	2	Correct prediction
3	EGFR substitution	3	3	Correct prediction
4	KRAS substitution	4	4	Correct prediction
5	TP53 deletion	5	5	Correct prediction
6	TP53 substitution	6	6	Correct prediction

**Table 24:** Output of the SVM ensemble on “unseen” HOG genomic samples.

S/N	Output class	Actual output	Target output	Remark
1	EGFR deletion	2	2	Correct prediction
2	EGFR substitution	6	3	Incorrect prediction
3	KRAS substitution	6	4	Incorrect prediction
4	TP53 deletion	5	5	Correct prediction
5	TP53 substitution	6	6	Correct prediction

**Table 25:** Nonensemble SVM result with varying kernel functions using LBP genomic samples.

S/N	Kernel function	MSE	Accuracy (%)
1	Linear	0.0945	43.2
2	Quadratic	0.0883	56.8
**3**	**Polynomial**	**0.0785**	**75.7**
4	RBF	0.0897	43.2
5	MLP	0.0871	18.9

**Table 26:** Confusion matrix of the nonensemble SVM with polynomial kernel function using LBP genomic samples.

a	b	c	d	e	f	Classified as
5	0	0	0	2	0	a = normal
0	7	0	0	0	0	b = EGFR deletion
0	0	5	0	0	0	c = EGFR substitution
0	0	0	5	0	0	d = KRAS substitution
0	0	0	0	6	0	e = TP53 deletion
0	0	0	0	7	0	f = TP53 substitution

**Table 27:** Output of the nonensemble SVM on “seen” LBP genomic samples.

S/N	Class name	Actual output	Target output	Remark
1	Normal			
	EGFR	1	1	Correct prediction
	KRAS	1	1	Correct prediction
	TP53	5	1	Incorrect prediction
2	EGFR deletion	2	2	Correct prediction
3	EGFR substitution	3	3	Correct prediction
4	KRAS substitution	1	4	Incorrect prediction
5	TP53 deletion	5	5	Correct prediction
6	TP53 substitution	5	6	Incorrect prediction

**Table 28:** Output of nonensemble SVM on “unseen” LBP genomic samples.

S/N	Output class	Actual output	Target output	Remark
1	EGFR deletion	2	2	Correct prediction
2	EGFR substitution	3	3	Correct prediction
3	KRAS substitution	4	4	Correct prediction
4	TP53 deletion	5	5	Correct prediction
5	TP53 substitution	5	6	Incorrect prediction

**Table 29:** Output of the SVM ensemble on “seen” LBP genomic samples.

S/N	Class name	Actual output	Target output	Remark
1	Normal			
	EGFR	1	1	Correct prediction
	KRAS	1	1	Correct prediction
	TP53	5	1	Incorrect prediction
2	EGFR deletion	2	2	Correct prediction
3	EGFR substitution	3	3	Correct prediction
4	KRAS substitution	1	4	Incorrect prediction
5	TP53 deletion	5	5	Correct prediction
6	TP53 substitution	5	6	Incorrect prediction

**Table 30:** Output of the SVM ensemble on “unseen” LBP genomic samples.

S/N	Output class	Actual output	Target output	Remark
1	EGFR deletion	2	2	Correct prediction
2	EGFR substitution	3	3	Correct prediction
3	KRAS substitution	4	4	Correct prediction
4	TP53 deletion	5	5	Correct prediction
5	TP53 substitution	1	6	Incorrect prediction

**Table 31:** Summary of experimental results.

S/N	Classifier/features extraction algorithm	Accuracy (%)	MSE
1	Nonensemble MLP-ANN/HOG	87.6	0.0355
2	MLP-ANN ensemble/HOG	95.9	0.0159
3	Nonensemble MLP-ANN/LBP	80.3	0.0530
4	MLP-ANN ensemble/LBP	82.4	0.0479
5	Nonensemble SVM/HOG	86.5	0.0706
6	SVM ensemble/HOG	91.9	0.0692
7	Nonensemble SVM/LBP	75.7	0.0785
8	SVM ensemble/LBP	75.7	0.0743
